# High prevalence of type 2 diabetes in Iraqi and Swedish residents in a deprived Swedish neighbourhood - a population based study

**DOI:** 10.1186/1471-2458-11-303

**Published:** 2011-05-12

**Authors:** Louise Bennet, Sven-Erik Johansson, Carl-David Agardh, Leif Groop, Jan Sundquist, Lennart Råstam, Kristina Sundquist

**Affiliations:** 1 Department of Clinical Sciences, Lund University, Malmö, Sweden; 2 Center for Primary Health Care Research, Region Skåne, Sweden

## Abstract

**Background:**

Immigrants from the Middle-East are at high risk of developing type 2 diabetes (T2D). The aim of the present survey was to measure, in a single deprived neighbourhood, the prevalence rates of impaired fasting glucose (IFG), impaired glucose tolerance (IGT) and T2D in residents originating from Iraq and to compare them to those in residents born in Sweden. An additional aim was to identify metabolic, lifestyle and socioeconomic risk factors associated with IFG/IGT and T2D in these residents.

**Methods:**

The study was conducted February 1'st to March 31'st 2010. Men and women aged 45 to 65 years of Swedish or Iraqi origin, living in the neighbourhood of Rosengård, Malmö, Sweden, were randomly selected from the census register. Each participant signed a written informed consent form, underwent a physical examination and an oral glucose tolerance test (OGTT), provided blood samples and filled in a questionnaire. A total of 175 subjects participated (Swedish origin *n *= 79, Iraqi origin *n *= 96), reflecting an overall response rate of almost 60%.

**Results:**

In total, 21.9% and 19.0% of the Iraqi and Swedish participants, respectively, suffered from T2D, while 24.0% of the Iraqi participants and 25.3% of the Swedish participants had IFG/IGT. There were no significant differences in prevalence rates relating to country of origin.

Obesity (BMI ≥30 kg/m^2^) and sedentary leisure time physical activity were highly prevalent in both groups, while a family history of diabetes was more prevalent in participants from Iraq (49.2%) than in those from Sweden (22.8%) (*p *= 0.001).

Being obese or having a sedentary leisure time were, independently associated with T2D (OR 5.43 (95% CI 2.10-14.02) and 2.89 (95% CI 1.03-8.10) respectively), while economic difficulties were independently associated with IFG/IGT (OR 2.55 (95% CI 1.06-6.15)) after adjustment for the confounding effects of other common risk factors for T2D.

**Conclusions:**

This study reveals a high prevalence of T2D, independently of country of origin (Iraq or Sweden), in a socially vulnerable area and additionally presents a risk factor profile that is markedly different from that of Sweden in general.

## Background

Type 2 diabetes (T2D), impaired fasting glucose (IFG) and impaired glucose tolerance (IGT) are strong risk factors for cardiovascular disease (CVD) and mortality [1,2]. The official prevalence rate of T2D in Sweden is 4% [3,4], but it has been suggested that the "true" prevalence is higher [5]. Globally, the Middle-East is highly affected by T2D with a prevalence rate varying between 7 and 22% [6,7]. In addition, migration may increase the risk of developing T2D [8-10]. Indeed, the prevalence of T2D in Sweden has been estimated to be 2-3 times higher amongst immigrants from the Middle-East as compared to inhabitants of Swedish origin [10]. However, the prevalence of IFG and IGT amongst immigrants from the Middle-East is unknown.

It is possible that adaptation to the culture of the country to which they have migrated - acculturation - may further increase the risk of developing T2D, as a consequence of the adoption of a sedentary lifestyle and a high intake of energy-dense food [11,12]. In addition, psychosocial and socioeconomic factors may further increase the risk of T2D and death in immigrant populations [13-15].

Immigrants tend to accumulate in deprived areas in large cities. Malmö is the third largest city in Sweden with almost 300,000 inhabitants in 2009 [16]. Of these, 30% are first- or second-generation immigrants. The largest immigrant group comprises Iraqis, who mainly live in the housing district of Rosengård. Rosengård is characterised by a high proportion of immigrants and high socioeconomic vulnerability. Earlier studies have shown that the neighbourhood socioeconomic environment is associated with the incidence of coronary heart disease [17] and prevalence of T2D [18]. The prevalence rates of T2D, IFG, IGT and risk factors for T2D are most likely higher in deprived and immigrant-dense neighbourhoods such as Rosengård, and more studies in such areas are needed in order to properly target preventive measures and screening instruments in high-risk populations.

The primary aim of this study was to examine the prevalence of IFG, IGT and T2D, and metabolic and lifestyle-associated risk factors for T2D, in residents in Rosengård originating from Iraq and Sweden. A second aim was to identify metabolic, lifestyle and socioeconomic risk factors associated with IFG/ IGT and T2D amongst the residents in this vulnerable area.

## Methods

### Subjects

The size of the population in Rosengård has changed little since the 1970's. In 2009, almost 22,000 people were living in Rosengård, of whom 60% originated from Iraq, the former Yugoslavia, Lebanon or Poland. The largest immigrant group in Rosengård comes from Iraq (almost 3,000 adult inhabitants).

**Figure 1:**
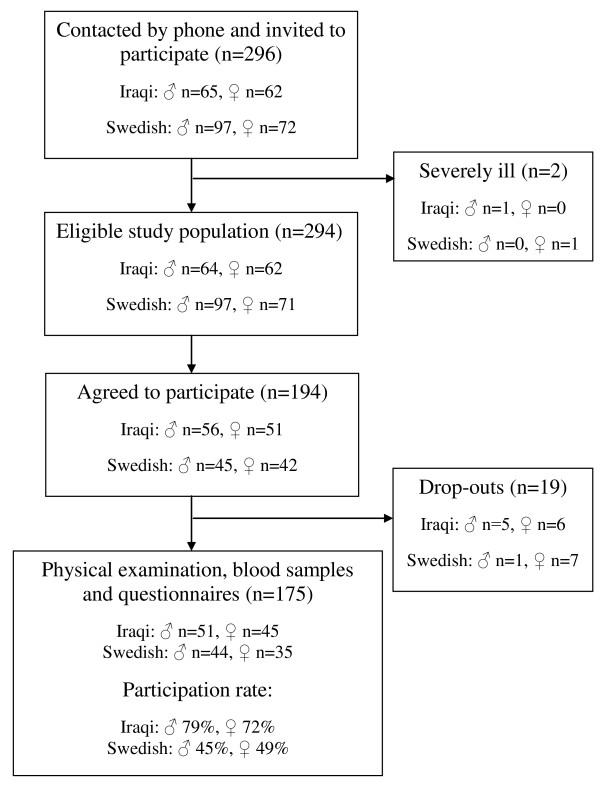
**Recruitment of the study population**. Inclusion criteria: men and women aged 45 to 65 years and living in Rosengård, Malmö. Exclusion criteria: severe mental or physical illness.

The present study aimed to recruit at least 158 subjects, with equal numbers of subjects of Iraqi and Swedish origin. This sample size was based on the following power calculation: a power of 80%, an alpha level of 5%, an estimated prevalence rate of T2D of 22.9% in residents from Iraq, and of 6.9% in residents from Sweden [10]. This gives a sample size of at least 79 participants each from Iraq and Sweden. A flow chart describing the study population, together with inclusion and exclusion criteria, is presented in Figure 1.

Residents in Rosengård aged 45 to 65 years were randomly selected from the census register and over the course of two months repeated attempts were made to contact them by phone to invite them to participate. Examinations were conducted between February 1 and March 31, 2010. Individuals agreeing to participate received a confirmation letter in the post and additional information, e.g., instructions to bring a list of their present medication and not to eat or drink later than 10 pm the day before testing. Participants underwent a physical examination and an OGTT, provided blood samples, and filled in a questionnaire.

### Care Need Index

The level of deprivation was estimated using the Care Need Index (CNI). The CNI is based on the socio-demographic characteristics of neighbourhoods, i.e., the proportions of elderly people living alone, children under the age of 5 years, residents who are unemployed, residents who have a low educational level, single parents, and residents born in Southern or Eastern Europe, Asia, Africa or South America, as well as rates of mobility [19]. It correlates with other instruments assessing neighbourhood deprivation such as the Townsend score and has been constructed so that the higher CNI score the more deprived the neighbourhood [19,20]. CNI scores have been calculated for all neighbourhoods in Sweden with >50 inhabitants. In 2007, the calculated CNI scores ranged from -52.5 (the most affluent neighbourhood) to 87.6, (the most deprived neighbourhood). The CNI score for Rosengård was 61.1, indicating that it is one of the most deprived neighbourhoods in Sweden.

### Assessment of clinical variables

Information on present medication, previous diagnosis of diabetes, hypertension and CVD, and family history of diabetes (in biological parents or siblings) were collected by nurses using structured forms with support from an Arabic-speaking interpreter when necessary. A standard physical examination was conducted by trained research nurses. It involved the measurement of blood pressure, weight, height, waist and hip circumferences, as well as the collection of blood samples and the performance of an OGTT.

*Blood pressure *was measured in the supine position after 5 minutes' rest with the arm at the level of the heart. The mean of two measurements, taken one minute apart, was calculated. The diagnosis of hypertension was based on previous diagnosis of hypertension by a physician or a systolic mean blood pressure of ≥ 140 mm Hg and/or a diastolic mean blood pressure of ≥ 90 mm Hg at the investigation [21].

*Height *was measured to the nearest cm.

*Weight *was measured to the nearest kg in subjects wearing light clothes, but not shoes.

*Waist circumference (WC) *was measured to the nearest cm in a standing position after a gentle expiration. A tape measure was placed around the bare midriff of each participant and the WC measured midway between the lower border of the rib cage and the superior border of the iliac crest [22].

*Abdominal obesity and body mass index (BMI) *Abdominal obesity was defined as waist circumference of ≥ 102 cm for males and ≥ 88 cm for females [23]. BMI (kg/m^2^) was calculated as weight (kg) divided by height (m) squared. Subjects were classified according to BMI using WHO definitions: normal weight: BMI < 25 kg/m^2^; overweight: BMI ≥ 25 kg/m^2 ^and < 30 kg/m^2^; and obese: BMI ≥ 30 kg/m^2^.

*Blood samples and oral glucose tolerance test (OGTT) *Blood samples were collected in the morning following an overnight 10-h fast. All blood samples were analyzed on-line during the study. Serum insulin levels were determined using the Radioimmunoassay technique (Access^© ^Ultrasensitive Insulin, Beckman Coulter, USA) [24]. Cholesterol and triglycerides in serum were analyzed using enzymatic methods (Bayer Diagnostics) [25]. HDL-cholesterol in serum was measured enzymatically after isolation of LDL and VLDL (Boehringer Mannheim GmbH, Germany) and LDL-cholesterol was estimated using Friedewald's method [26].

Then a standard 75-g OGTT was performed and further blood samples were collected at 0, 30, 60 and 120 min. Blood glucose was measured in capillary whole blood immediately after sampling by means of a HemoCue photometer (HemoCue AB, Ängelholm, Sweden) [27].

*Diabetes* was confirmed in subjects previously diagnosed with diabetes by a physician, as well as in new cases, by a fasting plasma glucose level of ≥ 7.0 mmol/L (measured twice) and/or by a 2-h plasma glucose level in the OGTT of ≥ 11.1 mmol/L [28]. Participants with previously confirmed diabetes did not undergo an OGTT. *Impaired fasting glucose (IFG) *was defined as a fasting plasma glucose level of ≥ 6.1 mmol/L and < 7.0 mmol/L and a 2-h plasma glucose level of < 7.8 mmol/L [28]. *Impaired glucose tolerance (IGT) *was defined as a fasting plasma glucose level of < 7.0 mmol/L and a 2-h plasma glucose level of ≥ 7.8 and < 11.1 mmol/L [28].

*Homeostasis model assessment (HOMA) *HOMA was used to estimate both insulin resistance (HOMA-IR) and beta cell function (HOMA-β) [29]. Indices were calculated as follows:

The insulin sensitivity index (ISI) was calculated from the OGTT [30] results as follows:

### Assessment of lifestyle and socioeconomic data

Information was collected by self-administered questionnaires in Swedish or Arabic, the latter translated by an authorised translator with Arabic as a native language, and with support from an Arabic-speaking interpreter when necessary.

*Leisure time physical activity (LTPA) *was classified as four categories: (1) sedentary LTPA: physical inactivity or less strenuous LTPA (walking, cycling, gardening, etc.), less than 2 hours a week (h/w); (2) moderate LTPA: less strenuous LTPA, more than 2 h/w; (3) strenuous LTPA: strenuous physical activity (jogging, swimming, tennis, etc.) of at least 30 minutes' duration, once or twice weekly; and (4) highly strenuous LTPA; strenuous physical activity of at least 30 minutes' duration, at least three times weekly [31]. Individuals in categories 2 to 4 were considered physically active in their leisure time while those in category 1 were considered sedentary.

*Smoking habits *Participants were categorised as non-smokers or active smokers. Non-smokers included never-smokers and participants that had stopped smoking more than 6 months ago [32].

*Alcohol intake *Participants were asked how many standard glasses of alcohol they usually consumed during an average week. One standard glass was equal to 50 centiliters (cL) of beer (3.5% alcohol by volume (abv)), 25 cL of strong beer (≥4.5% abv), 12-15 cL of wine (12-15% abv), 8 cL of wine (≥16 abv), or 4 cL of liquor [33]. Those consuming any amount of alcohol were considered alcohol consumers.

*Binge drinking *Men were asked how often during the last month they had consumed at least five standard glasses of alcohol on the same occasion, while women were asked how often they had consumed at least four standard glasses of alcohol on the same occasion [33].

*Educational level *was classified as three categories: (1) low (elementary school, up to 15 years of age), (2) intermediate (high school, 16 to 18 years of age) and (3) higher education (college/university, 18 years of age and older).

*Employment status *Participants were assigned to one of three categories: (1) active (employed, students, work experience), (2) non-active (including participants taking care of households, as well as those receiving activity compensation from the Swedish Employment Service) and (3) pensioner (retired due to age or illness).

*Economic difficulties *Difficulties in paying for food, rent or bills comprised three categories: (1) none and (2) once or (3) several times during the past year.

### Statistical analysis

The software used was SPSS 14.0 for Windows XP.

Differences in means between groups were adjusted for age using linear regression models (Additional file 1, Table 1). Differences in proportions between groups were adjusted for age using binary logistic regression models (when the dependent variable was binary, (Additional file 1, Tables 1 and 2) or multinomial logistic regression models (Additional file 1, Table 2).

Associations between diabetes-associated risk factors and IFG/IGT and T2D were estimated using multinomial logistic regression models with normal glucose metabolism as the reference. The associations between IFG/IGT and risk factors for T2D are presented in Additional file 2, Table 3a and associations between T2D and common risk factors for T2D in Additional file 2, Table 3b. Associations were expressed as odds ratios (ORs) with 95% confidence intervals (CIs). The 'goodness-of-fit' was tested using Pearson's goodness-of-fit test (*p *> 0.05 which indicated a good fit).

All tests were two-sided and a *p*-value of < 0.05 was considered statistically significant. First order interaction between family history of diabetes and country of origin was tested including their associations with IFG/IGT and T2D.

### Ethical considerations

The investigation conforms to the principles outlined in the Declaration of Helsinki [34]. The Ethics committee at Lund University approved the study (No. 2009/36) and written informed consent was given by all participants.

## Results

### Clinical characteristics of the study subjects

**Figure 2:**
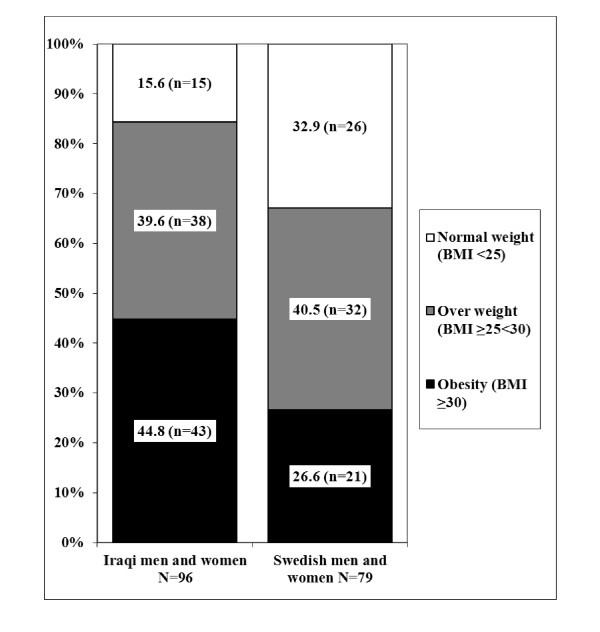
**BMI distribution of the study population**.

Characteristics of the study subjects are presented in Additional file 1, Table 1. Swedish participants were older on average than those of Iraqi origin, had higher systolic and diastolic blood pressure and were more frequently diagnosed with hypertension. On the other hand, they had a more favourable cholesterol risk factor profile, with higher levels of high density lipoproteins (HDL) and lower levels of triglycerides (TG) than the Iraqi participants. Sixteen percent of the Iraqi and 33% of the Swedish participants had normal body weights (Figure 2). Although obesity and abdominal obesity were highly prevalent in both groups, obesity was more prevalent in participants of Iraqi origin than in those of Swedish origin (OR 4.58 (95% CI 1.91-11.00); *p *= 0.001 vs. Swedish subjects). ISI scores were higher in the Swedish participants (Additional file 1, Table 1).

**Figure 3:**
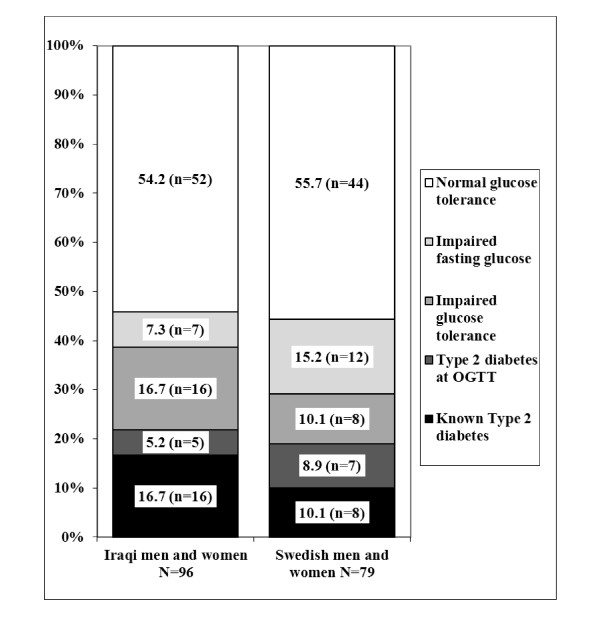
**Prevalence of type 2 diabetes (T2D), impaired glucose tolerance (IGT) and impaired fasting glucose (IFG) in participants of Iraqi and Swedish origin**.

The prevalence rates of T2D, IGT and IFG in all subjects are presented in Figure 3. OGTTs were performed in all participants (*n *= 151) except for 24 subjects (16 Iraqi and 8 Swedish) with previous diagnosis of diabetes. Approximately one third of the OGTT results were abnormal: 7.9% of participants (12/151) were diagnosed with T2D, 15.9% (24/151) with IGT and 12.6% (19/151) with IFG. There were no significant differences in the prevalence rates of T2D, IGT or IFG relating to country of origin: known T2D OR 2.37 (CI 0.88-6.36); T2D diagnosed at the OGTT OR 0.86 (CI 0.24-3.02); IGT OR 2.19 (CI 0.82-5.82); and IFG OR 0.50 (CI 0.18-1.44) (Figure 3).

Among obese participants, 76.2% (16/21) of those who were Swedish and 58.1% (25/43) of those who were Iraqi had T2D, IGT or IFG. IFG was less prevalent amongst obese Iraqi participants 7.0% (3/43), compared to obese Swedish participants 23.8% (5/21), OR 0.14 (CI 0.02-0.87). Otherwise, there were no significant differences in prevalence rates of T2D or IGT relating to country of origin. Although ISI scores were higher in the Swedish participants than in those from Iraq (Additional file 1, Table 1), the ISI scores did not differ between Iraqi and Swedish participants after adjustment for BMI (β-coefficient -9.47 (CI -29.05-10.11) with Swedish participants serving as the reference group.

Lifestyle and socioeconomic characteristics are presented in Additional file 1, Table 2. Sedentary LTPA was highly prevalent in both Iraqi and Swedish subjects. There were no significant differences in the prevalence of alcohol consumers relating to country of origin. However, binge drinking was more prevalent amongst Swedish participants compared to Iraqi participants. Iraqi men and women had a higher level of education than their Swedish counterparts. On the other hand, their employment status was more likely to be non-active and they more frequently reported economic difficulties.

### Risk factors independently associated with IFG, IGT and T2D

Associations between diabetes-associated risk factors and IFG/IGT are presented in Additional file 2, Table 3a and associations between diabetes-associated risk factors and T2D in Additional file 2, Table 3b. Having suffered economic difficulties was independently associated with IFG/IGT, while age, obesity and sedentary LTPA were independently associated with T2D. There were no interactions between the variables in the models.

## Discussion

### Principal findings

In this survey of residents of a deprived Swedish neighbourhood, 45.9% of the Iraqi subjects and 44.3% of the Swedish subjects had IFG, IGT or T2D. No significant differences in prevalence rates relating to origin were found. Economic difficulties were associated with IFG/IGT, while obesity and sedentary LTPA were highly prevalent and independently associated with T2D after adjustment for the confounding effects of age, sex, country of origin and family history of T2D.

### Prevalence of risk factors for T2D in Rosengård as compared to Sweden in general

The risk factor profile among individuals living in Rosengård, a deprived neighbourhood of Malmö, corresponds well with the findings of earlier studies showing that factors contributing to the risk of developing T2D such as obesity, sedentary lifestyle, psychosocial risk factors and low socioeconomic status tend to accumulate amongst immigrants [13-15,35,36]. However, our results also showed that non-immigrants living in the same area had a similar risk factor profile. In the age group studied, the prevalence rate of obesity was over two times higher than in the general Swedish population (13% in 2007) [16]. Obesity rates were higher in residents of Iraqi origin than in those of Swedish origin. This finding concurs with the results of another recent study of deprived neighbourhoods in an urban setting south of Stockholm in which the prevalence of obesity was found to be 42.9% in Middle-Eastern women and 16% in Swedish-born women [36]. In the present study the prevalence of sedentary LTPA was about five times higher in this deprived neighbourhood than in Sweden in general (12%) [16]. Although the educational level was higher amongst the Iraqi residents than amongst the Swedish ones, Iraqis were more likely to be non-active. Iraqi residents also frequently reported economic difficulties, their prevalence being twice as high as has been reported among foreign-born inhabitants in Sweden in general (44% vs. 22%) [16]. Additionally, economic difficulties were almost three times more common amongst Swedish residents in Rosengård than in Sweden in general in the same age group (22% vs. 8.6%) [16], which together with the high CNI value reflects the socioeconomic deprivation of the area.

### Risk factors influencing T2D amongst Iraqi and Swedish subjects

Obesity and sedentary LTPA were highly prevalent in both Iraqi and Swedish participants. These data are consistent with those from other studies of vulnerable areas [36], but also presents a risk factor profile that is markedly different from Sweden in general.

Obesity and sedentary lifestyle are well established predictors of T2D [37,38] and were, in this survey, strongly associated with T2D. Immigrants from the Middle-East have been identified as being at high risk of developing T2D [8,10]. In this survey, the prevalence rate of T2D in residents originating from Iraq corresponded well with prevalence rates reported in earlier studies of Middle-Eastern immigrants in Sweden [10]. However, the prevalence rate of T2D was three times higher in Iraqi as well as in Swedish study participants as compared to the prevalence rates reported from Iraq (7.4 to 7.8%) [6,7] and in Sweden in general (7.3%) [6].

The 21 Iraqis diagnosed with T2D had lived in Sweden for an average of 11.8 years (range 7 to 20 years) and had been diagnosed with T2D an average of 5.8 years earlier (range 0 to 35 years). Thus most of them had developed T2D in Sweden. Our results indicate that residents in Rosengård seem to be at higher risk for T2D than those living in other parts of Sweden, independently of ethnic background, and correspond well with previous studies that reported that the neighbourhood socioeconomic environment has a strong impact on the development of T2D [18].

Iraqi subjects with T2D are less likely to be insulin-resistant and more likely to display beta cell dysfunction than Swedish subjects with T2D [8], suggesting that genetic factors may have a greater influence on the risk of T2D in Iraqis [8]. The ISI scores recorded in this survey were higher in Swedish men and women than in Iraqis, independently of age. However, this was probably due to the lower average BMI in the Swedish participants. After adjustment for BMI, these differences did not remain.

Heredity for T2D, which increases the risk for T2D [37], is reported to be more prevalent amongst immigrants from the Middle-East than in Swedish subjects [8]. In the present study, a family history of T2D was more prevalent in Iraqi residents than in Swedish ones. However, having a family history of T2D did not seem to increase the risk for IFG/IGT or T2D (Additional file 2, Tables 3a and 3b). The strong accumulation of risk factors for T2D represents a risk factor profile that is markedly different from that of Sweden in general. Our results indicate that the risk factor profile for T2D differs according to setting. Consequently, the influence of confounding by obesity and sedentary LTPA is probably present and the high prevalence of these risk factors may have attenuated the effects of other well known risk factors for T2D such as country of origin and heredity for T2D. However, the sample size was rather small, and may be the reason for the non-significant effects of other traditional T2D risk factors.

Socioeconomic status is associated with health [39]. Economic difficulties were prevalent and independently associated with IFG and IGT, indicating the impact of poor socioeconomic status on the risk of developing impaired glucose metabolism. A large proportion of the Iraqi participants were well educated but at the same time "non-active". Therefore, there is a potential to decrease the proportion who are unemployed in order to improve their socioeconomic situation and consequently promote better health.

### Clinical implications

This study led us to determine not only that a large proportion of participants were suffering from undetected T2D but also that a large proportion of them were at high risk of developing T2D over the coming years (i.e., individuals identified with IFG or IGT). Although different genetic and environmental factors may trigger the development of T2D in Iraqi and Swedish residents, most individuals living in this neighbourhood displayed one or more lifestyle-associated risk factors for T2D.

Studies have shown that 20% of individuals with obesity will develop T2D within 10-years of diagnosis and 50% of those with early signs of diabetes will develop T2D during the same period of time [40]. Lifestyle interventions can reduce the incidence of diabetes by half, and studies have also shown that these interventions are cost-effective for society [41-43]. Thus, efforts in primary health care and other health care settings with the purpose to identify individuals at high risk of developing T2D and to support their efforts to adopt and sustain a health-promoting lifestyle are most likely worthwhile.

### Strengths and limitations

A key strength of this study is the study design: a population based survey of residents of Swedish and Iraqi origin living in a deprived neighbourhood who were randomly selected from the census register. The participation rate corresponded well with that of a population-based study conducted in another deprived Swedish neighbourhood [36]. Nonetheless, the high non-response rate may have introduced a selection bias. Swedish men and women had a lower participation rate which may be due to their higher rates of employment (the health examinations were conducted on weekday mornings without financial compensation).

We do not have information concerning lifestyle-associated risk factors and T2D in non-participants, which is a limitation. Another limitation concerns the relatively small study population, which may have influenced our findings. Additionally, a large proportion of the Iraqi participants declined to answer the questions concerning alcohol consumption. Finally, reporting of family history of T2D may suffer from recall bias, partly since it may be difficult to recall diseases suffered by parents and siblings, but also because many participants came from war-torn-areas and have broken families.

## Conclusions

The high accumulation of risk factors for T2D in both immigrants and non-immigrants living in Rosengård represents a risk factor profile that is markedly different from that in Sweden in general. The high prevalence of risk factors for T2D probably attenuated the effect of other risk factors such as family history of T2D as well as country of origin, but nevertheless indicates the strong influence of socioeconomic and lifestyle-associated risk factors in this residential area. Larger prospective surveys are needed to identify the genetic and lifestyle-associated mechanisms that trigger the development of T2D in relation to socioeconomic environment and ethnic background.

In addition, efforts in health care settings with the purpose to identify individuals in deprived areas at high risk of developing T2D are most likely worthwhile.

## Abbreviations

abv: alcohol by volume; BMI: body mass index; CI: confidence interval; cL: centilitres; conc.: concentration; CNI: care need index; CVD: cardiovascular disease; DBP: diastolic blood pressure; f: fasting; glc: glucose; HDL: high density lipoprotein; HOMA-β: homeostasis model assessment β-cell function; HOMA-IR: homeostasis model assessment insulin resistance; IFG: impaired fasting glucose; IGT: impaired glucose tolerance; ISI: insulin sensitivity index; LDL: low density lipoprotein; LTPA: leisure time physical activity; OGTT: oral glucose tolerance test; OR: odds ratio; SBP: systolic blood pressure; TG: triglycerides; T2D: type 2 diabetes

## Competing interests

The authors declare that they have no competing interests.

## Authors' contributions

LB participated in the design of the study and planned and organised its delivery, collected the data, performed the statistical analysis, analysed and interpreted the data and drafted the manuscript. SEJ participated in the statistical analysis, interpretation of the data and drafting of the manuscript. CDA was involved in the analysis and interpretation of the data and drafting of the manuscript. LG was involved in the design of the study, analysis and interpretation of the data and drafting of the manuscript. JS, LR and KS were involved in analysing and interpreting the data and drafting the manuscript. All authors read and approved the final manuscript.

## Pre-publication history

The pre-publication history for this paper can be accessed here:

http://www.biomedcentral.com/1471-2458/11/303/prepub

## Supplementary Material

Additional file 1 File 1: Tables 1 and 2. Click here for file

Additional file 2 File 2: Tables 3a and 3b. Click here for file

## References

[B1] Haffner SM Lehto S Ronnemaa T Pyorala K Laakso M Mortality from coronary heart disease in subjects with type 2 diabetes and in nondiabetic subjects with and without prior myocardial infarction N Engl J Med 1998 339 4 229 234 10.1056/NEJM199807233390404 9673301

[B2] Unwin N Shaw J Zimmet P Alberti KG Impaired glucose tolerance and impaired fasting glycaemia: the current status on definition and intervention Diabet Med 2002 19 9 708 723 12207806 10.1046/j.1464-5491.2002.00835.x

[B3] Berger B Stenstrom G Sundkvist G Incidence, prevalence, and mortality of diabetes in a large population. A report from the Skaraborg Diabetes Registry Diabetes Care 1999 22 5 773 778 10.2337/diacare.22.5.773 10332680

[B4] Jansson SP Andersson DK Svardsudd K Prevalence and incidence rate of diabetes mellitus in a Swedish community during 30 years of follow-up Diabetologia 2007 50 4 703 710 10.1007/s00125-007-0593-4 17268796

[B5] Lyssenko V Almgren P Anevski D Perfekt R Lahti K Nissen M Isomaa B Forsen B Homstrom N Saloranta C Predictors of and longitudinal changes in insulin sensitivity and secretion preceding onset of type 2 diabetes Diabetes 2005 54 1 166 174 10.2337/diabetes.54.1.166 15616025

[B6] International diabetes federation http://www.eatlas.idf.org/

[B7] Mansour AA Wanoose HL Hani I Abed-Alzahrea A Diabetes screening in Basrah, Iraq: a population-based cross-sectional study Diabetes Res Clin Pract 2008 79 1 147 150 10.1016/j.diabres.2007.07.016 17767973

[B8] Glans F Elgzyri T Shaat N Lindholm E Apelqvist J Groop L Immigrants from the Middle-East have a different form of Type 2 diabetes compared with Swedish patients Diabet Med 2008 25 3 303 307 10.1111/j.1464-5491.2007.02366.x 18307458

[B9] Sundquist J Winkleby MA Cardiovascular risk factors in Mexican American adults: a transcultural analysis of NHANES III, 1988-1994 Am J Public Health 1999 89 5 723 730 10.2105/AJPH.89.5.723 10224985 PMC1508740

[B10] Wandell PE Johansson SE Gafvels C Hellenius ML de Faire U Sundquist J Estimation of diabetes prevalence among immigrants from the Middle East in Sweden by using three different data sources Diabetes Metab 2008 34 4 Pt 1 328 333 18539497 10.1016/j.diabet.2008.01.012

[B11] Dixon LB Sundquist J Winkleby M Differences in energy, nutrient, and food intakes in a US sample of Mexican-American women and men: findings from the Third National Health and Nutrition Examination Survey, 1988-1994 Am J Epidemiol 2000 152 6 548 557 10.1093/aje/152.6.548 10997545

[B12] Neuhouser ML Thompson B Coronado GD Solomon CC Higher fat intake and lower fruit and vegetables intakes are associated with greater acculturation among Mexicans living in Washington State J Am Diet Assoc 2004 104 1 51 57 10.1016/j.jada.2003.10.015 14702584

[B13] Gadd M Sundquist J Johansson SE Wandell P Do immigrants have an increased prevalence of unhealthy behaviours and risk factors for coronary heart disease? Eur J Cardiovasc Prev Rehabil 2005 12 6 535 541 10.1097/00149831-200512000-00004 16319542

[B14] Nilsson PM Johansson SE Sundquist J Low educational status is a risk factor for mortality among diabetic people Diabet Med 1998 15 3 213 219 10.1002/(SICI)1096-9136(199803)15:3<213::AID-DIA569>3.0.CO;2-# 9545122

[B15] Robinson N Lloyd CE Stevens LK Social deprivation and mortality in adults with diabetes mellitus Diabet Med 1998 15 3 205 212 10.1002/(SICI)1096-9136(199803)15:3<205::AID-DIA519>3.0.CO;2-# 9545121

[B16] Statistics Sweden http://www.scb.se Statistics Sweden

[B17] Sundquist K Winkleby M Ahlen H Johansson SE Neighborhood socioeconomic environment and incidence of coronary heart disease: a follow-up study of 25,319 women and men in Sweden Am J Epidemiol 2004 159 7 655 662 10.1093/aje/kwh096 15033643

[B18] Evans JM Newton RW Ruta DA MacDonald TM Morris AD Socio-economic status, obesity and prevalence of Type 1 and Type 2 diabetes mellitus Diabet Med 2000 17 6 478 480 10.1046/j.1464-5491.2000.00309.x 10975218

[B19] Malmstrom M Sundquist J Bajekal M Johansson SE Indices of need and social deprivation for primary health care Scand J Soc Med 1998 26 2 124 130 9658512 10.1177/14034948980260021301

[B20] Sundquist K Malmstrom M Johansson SE Sundquist J Care Need Index, a useful tool for the distribution of primary health care resources J Epidemiol Community Health 2003 57 5 347 352 10.1136/jech.57.5.347 12700218 PMC1732439

[B21] 1999 World Health Organization-International Society of Hypertension Guidelines for the Management of Hypertension. Guidelines Subcommittee J Hypertens 1999 17 2 151 183 10067786

[B22] Clinical Guidelines on the Identification, Evaluation, and Treatment of Overweight and Obesity in Adults--The Evidence Report National Institutes of Health Obes Res 1998 6 Suppl 2 51S 209S 9813653

[B23] Yusuf S Hawken S Ounpuu S Dans T Avezum A Lanas F McQueen M Budaj A Pais P Varigos J Effect of potentially modifiable risk factors associated with myocardial infarction in 52 countries (the INTERHEART study): case-control study Lancet 2004 364 9438 937 952 10.1016/S0140-6736(04)17018-9 15364185

[B24] Thorell J Larson SM Radioimmunoassay and related techniques The CV Mosby Company, ST Louis 1978 205 211

[B25] Allain CC Poon LS Chan CS Richmond W Fu PC Enzymatic determination of total serum cholesterol Clin Chem 1974 20 4 470 475 4818200

[B26] Friedewald WT Levy RI Fredrickson DS Estimation of the concentration of low-density lipoprotein cholesterol in plasma, without use of the preparative ultracentrifuge Clin Chem 1972 18 6 499 502 4337382

[B27] von Schenck H Falkensson M Lundberg B Evaluation of "HemoCue," a new device for determining hemoglobin Clin Chem 1986 32 3 526 529 3948400

[B28] World Health Organization Definition, diagnosis and classification of diabetes mellitus and its complications: Report of a WHO consultation. Part 1. Diagnosis and classification of diabetes mellitus 1999 Geneva, World Health Organization

[B29] Matthews DR Hosker JP Rudenski AS Naylor BA Treacher DF Turner RC Homeostasis model assessment: insulin resistance and beta-cell function from fasting plasma glucose and insulin concentrations in man Diabetologia 1985 28 7 412 419 10.1007/BF00280883 3899825

[B30] Matsuda M DeFronzo RA Insulin sensitivity indices obtained from oral glucose tolerance testing: comparison with the euglycemic insulin clamp Diabetes Care 1999 22 9 1462 1470 10.2337/diacare.22.9.1462 10480510

[B31] Sepp H Ekelund U Becker W Enkätfrågor om kost och fysisk aktivitet bland vuxna Livsmedelsverket 2004 rapport 21

[B32] Bakkevig O Steine S von Hafenbradl K Laerum E Smoking cessation. A comparative, randomised study between management in general practice and the behavioural programme SmokEnders Scand J Prim Health Care 2000 18 4 247 251 10.1080/028134300448832 11205095

[B33] Saunders JB Aasland OG Babor TF de la Fuente JR Grant M Development of the Alcohol Use Disorders Identification Test (AUDIT): WHO Collaborative Project on Early Detection of Persons with Harmful Alcohol Consumption--II Addiction 1993 88 6 791 804 10.1111/j.1360-0443.1993.tb02093.x 8329970

[B34] WMA Declaration of Helsinki - Ethical principles of medical research involving human subjects 2008 http://www.wma.net/en/30publications/10policies/b3/24964504

[B35] van Dieren S Beulens JW van der Schouw YT Grobbee DE Neal B The global burden of diabetes and its complications: an emerging pandemic Eur J Cardiovasc Prev Rehabil 17 Suppl 1 S3 8 10.1097/01.hjr.0000368191.86614.5a 20489418

[B36] Faskunger J Eriksson U Johansson SE Sundquist K Sundquist J Risk of obesity in immigrants compared with Swedes in two deprived neighbourhoods BMC Public Health 2009 9 304 10.1186/1471-2458-9-304 19698119 PMC2748077

[B37] Lyssenko V Jonsson A Almgren P Pulizzi N Isomaa B Tuomi T Berglund G Altshuler D Nilsson P Groop L Clinical risk factors, DNA variants, and the development of type 2 diabetes N Engl J Med 2008 359 21 2220 2232 10.1056/NEJMoa0801869 19020324

[B38] Manson JE Rimm EB Stampfer MJ Colditz GA Willett WC Krolewski AS Rosner B Hennekens CH Speizer FE Physical activity and incidence of non-insulin-dependent diabetes mellitus in women Lancet 1991 338 8770 774 778 10.1016/0140-6736(91)90664-B 1681160

[B39] Marmot M Friel S Bell R Houweling TA Taylor S Closing the gap in a generation: health equity through action on the social determinants of health Lancet 2008 372 9650 1661 1669 10.1016/S0140-6736(08)61690-6 18994664

[B40] Tuomilehto J Nonpharmacologic therapy and exercise in the prevention of type 2 diabetes Diabetes Care 2009 32 Suppl 2 S189 193 19875550 10.2337/dc09-S308 PMC2811487

[B41] Hoerger TJ Hicks KA Sorensen SW Herman WH Ratner RE Ackermann RT Zhang P Engelgau MM Cost-effectiveness of screening for pre-diabetes among overweight and obese U.S. adults Diabetes Care 2007 30 11 2874 2879 10.2337/dc07-0885 17698614

[B42] Knowler WC Barrett-Connor E Fowler SE Hamman RF Lachin JM Walker EA Nathan DM Reduction in the incidence of type 2 diabetes with lifestyle intervention or metformin N Engl J Med 2002 346 6 393 403 11832527 10.1056/NEJMoa012512 PMC1370926

[B43] Tuomilehto J Lindstrom J Eriksson JG Valle TT Hamalainen H Ilanne-Parikka P Keinanen-Kiukaanniemi S Laakso M Louheranta A Rastas M Prevention of type 2 diabetes mellitus by changes in lifestyle among subjects with impaired glucose tolerance N Engl J Med 2001 344 18 1343 1350 10.1056/NEJM200105033441801 11333990

