# Supplementary material for: High prevalence of type 2 diabetes in Iraqi and Swedish residents in a deprived Swedish neighbourhood - a population based study
Source: BMC Public Health. 2011 May 12;11:303. doi: 10.1186/1471-2458-11-303 (PMC3120683; doi:10.1186/1471-2458-11-303)
Supplement: Additional file 1 — File 1: Tables 1 and 2. [file 1471-2458-11-303-S1.DOC]

**Table 1. Clinical characteristics (means, percentages and numbers) of the study population**

| **Variable** | **Country of origin**  **Iraq Sweden**  ***N*=96 *N*=79** | | **β-coefficient /**  **OR (95% CI)** |
| --- | --- | --- | --- |
| Age (years) | 54.3 | 56.8 | -2.48 (-4.17 to-0.79)^**^ |
| Male sex, % (n) | 53.1 (51) | 55.7 (44) | 0.94 (0.51 to 1.74) |
| SBP (mmHg) | 132 | 140 | -7.17(-12.96 to-1.38)^*^ |
| DBP (mmHg) | 82 | 87 | -4.21 (-7.78 to-0.64)^*^ |
| BMI (kg/m^2^) | 29.5 | 27.6 | 2.40 (0.91 to 3.89)^**^ |
| Total cholesterol (mmol/L) | 5.1 | 5.4 | -0.24 (-0.56 to 0.08) |
| LDL (mmol/L) | 3.4 | 3.6 | -0.17 (-0.45 to 0.11) |
| HDL (mmol/L) | 1.0 | 1.2 | -0.19 (-0.30 to-0.08)^***^ |
| TG (mmol/L) | 1.6 | 1.3 | 0.28 (0.02 to 0.55)^*^ |
| HOMA-IR | 3.9 | 2.4 | 0.74 (-0.14 to 1.62) |
| HOMA-β | 87.5 | 76.7 | 7.50 (-10.80 to 25.82) |
| ISI | 98.1 | 119.3 | -22.68 (-44.18 to-1.19)^*^ |
| Abdominal obesity, % (n) | 65.3^a^ (62) | 58.2 (46) | 1.43 (0.75 to 2.70) |
| - Missing % (n) | 1.0 (1) |  |  |
| Family history T2D, % (n) | 49.0 (47) | 22.8 (18) | 3.00 (1.53 to 5.88)^***^ |
| Hypertension, %^a^ (n)^b^ | 41.7 (40) | 64.6 (51) | 0.49 (0.26 to 0.95)^**^ |
| CVD, % (n)^b,c^ | 11.4 (11) | 11.4 (9) | 1.15 (0.44 to 2.99) |

Differences in means between groups were adjusted for age using linear regression models and expressed as β-coefficients with 95% CIs. All tests were two-sided and a *p*-value of <0.05 was considered statistically significant. Differences in proportions between groups were adjusted for age using binary logistic regression models and expressed as ORs with 95% CI. ^*^*p*<0.05, ^**^*p*<0.01, ^***^*p*<0.001.

^a^ Valid percent

^b^New and previously diagnosed cases.

^c^History of ischaemic cardiovascular disease, congestive heart failure and/or stroke.

**Table 2. Lifestyle and socioeconomic characteristics (percentages and numbers) of the study population**

| **Variable % (n)** | **Country of origin**  **Iraq Sweden *N*=96 *N*=79** | | **OR (95% CI)** |
| --- | --- | --- | --- |
| Sedentary LTPA | 71.3^a^ (62) | 61.5^a^ (48) | 1.37 (0.70–2.67) |
| - Missing | 9.4 (9) | 1.3 (1) |  |
| Tobacco consumption | 31.2^a^ (29) | 37.2^a^ (29) | 0.67 (0.34–1.30) |
| - Missing | 3.1 (3) | 1.3 (1) |  |
| Alcohol consumption | 50.0^a^ (7) | 69.6^a^ (48) | 0.60 (0.17–2.10) |
| - Missing | 85.4 (82) | 12.7 (10) |  |
| Binge drinking | 8.1^a^ (5) | 36.5^a^ (19) | 0.15 (0.05–0.46)^***^ |
| - Missing | 35.4 (34) | 34.2 (27) |  |
| Education level |  |  |  |
| - High | 53.7^a^ (51) | 21.8^a^ (17) | 1.00 |
| - Intermediate | 16.8^a^ (16) | 42.3^a^ (33) | 0.16 (0.07–0.36)^***^ |
| - Low | 29.5^a^ (28) | 35.9^a^ (28) | 0.34 (0.16–0.74)^**^ |
| - Missing | 1.0 (1) | 1.3 (1) |  |
| Employment status |  |  |  |
| - Active | 49.4^a^ (43) | 65.8^a^ (48) | 1.00 |
| - Pensioner | 9.2^a^ (8) | 11.0^a^ (8) | 1.50 (0.50–4.54) |
| - Non-active | 41.4^a^ (36) | 23.3^a^ (17) | 2.52 (1.21–5.22)^*^ |
| - Missing | 9.4 (9) | 7.6 (6) |  |
| Economic difficulties on one or several occasions | 44.0^a^ (40) | 21.8^a^ (17) | 2.59 (1.29–5.17)^**^ |
| - Missing | 5.2 (5) | 1.3 (1) |  |

Differences in proportions between groups were adjusted for age using binary logistic regression models when the dependent variable was binary otherwise multinomial regression models were used (in the variables “education level” and “employment status”).

Differences were expressed as ORs with 95% CI. ^*^*p*<0.05, ^**^*p*<0.01, ^***^*p*<0.001.

^a^ Valid percent
