# Supplementary material for: High prevalence of type 2 diabetes in Iraqi and Swedish residents in a deprived Swedish neighbourhood - a population based study
Source: BMC Public Health. 2011 May 12;11:303. doi: 10.1186/1471-2458-11-303 (PMC3120683; doi:10.1186/1471-2458-11-303)
Supplement: Additional file 2 — File 2: Tables 3a and 3b. [file 1471-2458-11-303-S2.DOC]

**Table 3a. Associations between impaired fasting glucose/impaired glucose tolerance and risk factors for T2D.**

| **Risk factors** | **Model I**  **OR (95% CI)** | | | **Model II**  **OR (95% CI)** | **Model III**  **OR (95% CI)** | **Model IV**  **OR (95% CI)** | **Model V**  **OR (95% CI)** |
| --- | --- | --- | --- | --- | --- | --- | --- |
| Age | 1.06 (0.99–1.13) | | | 1.06 (0.99–1.14) | 1.05 (0.98–1.12) | 1.04 (0.97–1.13) | 1.06 (0.98–1.15) |
| Sex |  | | |  |  |  |  |
| - Female | 1.00 | | | 1.00 | 1.00 | 1.00 | 1.00 |
| - Male | 1.91 (0.91–4.01) | | | 2.08 (0.96–4.51) | 2.06 (0.94–4.50) | 2.29 (0.97–5.43) | 2.26 (0.94–5.47) |
| Country of origin |  | | |  |  |  |  |
| - Sweden | 1.00 | | | 1.00 | 1.00 | 1.00 | 1.00 |
| - Iraq | 1.16 (0.54–2.46) | | | 1.06 (0.48–2.32) | 0.94 (0.42–2.10) | 0.69 (0.29–1.67) | 0.59 (0.24–1.49) |
| Family history of T2D^a^ | |  | |  |  |  |  |
| - No |  | | | 1.00 | 1.00 | 1.00 | 1.00 |
| - Yes |  | | | 1.43 (0.63–3.24) | 1.21 (0.52–2.81) | 1.30 (0.52–3.26) | 1.24 (0.48–3.19) |
| BMI |  | | |  |  |  |  |
| - <30 kg/m^2^ |  | | |  | 1.00 | 1.00 | 1.00 |
| - >30 kg/m^2^ |  | | |  | 2.20 (0.97–4.95) | 2.05 (0.85–4.95) | 2.22 (0.90–5.49) |
| Sedentary LTPA |  | | |  |  |  |  |
| - No |  | | |  |  | 1.00 | 1.00 |
| - Yes |  | | |  |  | 1.75 (0.72–4.24) | 1.62 (0.65–4.01) |
| Economic difficulties | | |  |  |  |  |  |
| - No |  | | |  |  |  | 1.00 |
| - Yes |  | | |  |  |  | 2.55 (1.06–6.15)^*^ |

Data were analysed using multinomial logistic regression models. Tables 3a and 3b are both based on the same analysis. This table presents the results for IFG/IGT with normal glucose metabolism as the reference. The covariates (risk factors) were included stepwise in the model. Associations are expressed as odds ratios (ORs) with 95% confidence intervals (CIs). ^*^*p*<0.05.

^a^ In biological parents and/or siblings

**Table 3b. Associations between T2D and risk factors for T2D**

| **Risk factors** | **Model I**  **OR (95% CI)** | | | **Model II**  **OR (95% CI)** | | **Model III**  **OR (95% CI)** | **Model IV**  **OR (95% CI)** | **Model V**  **OR (95% CI)** |
| --- | --- | --- | --- | --- | --- | --- | --- | --- |
| Age (years) | 1.14 (1.06–1.23)^***^ | | | 1.14 (1.06–1.23)^***^ | | 1.12 (1.03–1.22)^**^ | 1.14 (1.04–1.25)^**^ | 1.14 (1.04–1.25)^**^ |
| Sex |  | | |  | |  |  |  |
| - Female | 1.00 | | | 1.00 | | 1.00 | 1.00 | 1.00 |
| - Male | 1.97 (0.87–4.65) | | | 2.02 (0.86–4.70) | | 2.02 (0.82–4.94) | 1.81 (0.71–4.62) | 1.80 (0.70–4.63) |
| Country of origin |  | | |  | |  |  |  |
| - Sweden | 1.00 | | | 1.00 | | 1.00 | 1.00 | 1.00 |
| - Iraq | 1.16 (0.54–2.46) | | | 1.60 (0.68–3.76) | | 1.18 (0.48–2.92) | 1.08 (0.42–2.76) | 1.11 (0.42–2.98) |
| Family history of T2D^a^ | | |  |  | |  |  |  |
| - No |  | | | 1.00 | | 1.00 | 1.00 | 1.00 |
| - Yes |  | | | 1.11 (0.45–2.76) | | 0.77 (0.29–2.07) | 0.71 (0.25–1.99) | 0.65 (0.23–1.84) |
| BMI (kg/m^2^) |  | | |  | |  |  |  |
| - <30 |  | | |  | | 1.00 | 1.00 | 1.00 |
| - >30 |  | | |  | | 5.45 (2.21–13.46)^***^ | 5.25 (2.06–13.41)^***^ | 5.43 (2.10–14.02)^***^ |
| Sedentary LTPA |  | | |  | |  |  |  |
| - No |  | | |  | |  | 1.00 | 1.00 |
| - Yes |  | | |  | |  | 3.01 (1.08–8.39)^*^ | 2.89 (1.03–8.10)^*^ |
| Economic difficulties | |  | |  | |  |  |  |
| - No |  | | |  | |  |  | 1.00 |
| - Yes |  | | |  |  |  | | 1.24 (0.45–3.38) |

Data were analysed using multinomial logistic regression models. Tables 3a and 3b are both based on the same analysis. This table presents the results for T2D with normal glucose metabolism as the reference. The covariates (risk factors) were included stepwise in the model. Associations are expressed as ORs with 95% CIs. ^*^*p*<0.05, ^**^*p*<0.01, ^***^*p*<0.001.

^a^ In biological parents and/or siblings.
